# Higher adiponectin concentrations are associated with reduced metabolic syndrome risk independently of weight status in Brazilian adolescents

**DOI:** 10.1186/s13098-019-0435-9

**Published:** 2019-05-24

**Authors:** Karen Sparrenberger, Mariana Sbaraini, Felipe Vogt Cureau, Gabriela Heiden Teló, Luciana Bahia, Beatriz D. Schaan

**Affiliations:** 1 Postgraduate Program in Endocrinology, Universidade Federal do Rio Grande do Sul-Hospital de Clínicas de Porto Alegre, Rua Ramiro Barcelos 2350, Prédio 21, 6º andar, Porto Alegre, RS 90035-003 Brazil; 2 Postgraduate Program in Cardiology, Universidade Federal do Rio Grande do Sul, Porto Alegre, Brazil; 3 National Institute of Science and Technology for Health Technology Assessment (IATS), Hospital de Clínicas de Porto Alegre, Porto Alegre, Brazil; 4 Endocrine Division, Hospital de Clínicas de Porto Alegre, Porto Alegre, Brazil

**Keywords:** Adiponectin, Metabolic syndrome, Adolescents, Pediatric, Obesity

## Abstract

**Objective:**

To evaluate the association between adiponectin concentrations and metabolic syndrome (MetS) risk and to investigate if this association is independent of weight status in adolescents.

**Methods:**

Adiponectin concentrations and MetS risk were assessed in 4546 Brazilian adolescents (12–17 years old) enrolled in The Study of Cardiovascular Risks in Adolescents (“ERICA”), a cross-sectional multicenter study in Brazil. For analyses, adiponectin was categorized in sex and age-specific quartiles and MetS risk was expressed as a continuous score, calculated as the average of the standardized values (z-score) of the five MetS components. Multiple linear regression models were used to investigate the association between the quartiles of adiponectin and MetS risk.

**Results:**

Adiponectin was inversely associated with waist circumference and log-transformed triglycerides, and positively associated with HDL-c. We also observed an inverse association between adiponectin concentrations and MetS risk. After adjustment for sociodemographic variables, physical activity, skipping breakfast and body mass index (BMI), higher quartiles of adiponectin remained inversely associated with waist circumference and MetS risk. A direct association between adiponectin and HDL-c was also observed. In further analysis, the sample was stratified by weight status and an inverse association between quartiles of adiponectin and MetS risk was observed in both normal weight and overweight/obese adolescents.

**Conclusion:**

Higher adiponectin concentrations were independently and inverse associated with MetS risk in Brazilian adolescents, even after adjusting for BMI. These results were similar in normal weight and overweight/obese adolescents, suggesting that adiponectin may play a role in early development of MetS.

**Electronic supplementary material:**

The online version of this article (10.1186/s13098-019-0435-9) contains supplementary material, which is available to authorized users.

## Introduction

Metabolic syndrome (MetS) is a constellation of classical cardio-metabolic risk factors, including abdominal obesity, hypertension, dyslipidemia and hyperglycemia [1, 2]. The prevalence of MetS in the pediatric population has increased considerably in recent years and it has been associated with an increased risk for development of cardiovascular diseases during life [3, 4].

The etiology of MetS is complex and still not completely elucidated, thus different mechanisms remain under investigation [5]. In this context, adipokines have been studied as potential factors that play a role in the development of MetS [6, 7]. One of the most studied adipokines is adiponectin, which is produced in abundance by adipose tissue and is responsible for modulation of several metabolic processes, such as glucosec homeostasis and oxidation of fatty acids. In addition, adiponectin also has anti-inflammatory properties [8, 9].

In a previous publication, we described the adiponectin distribution and showed that lower levels of this adipokine were associated with general and abdominal obesity among Brazilian adolescents [10]. In metabolically unhealthy subjects, especially in the presence of obesity, circulating adiponectin is also reduced [11–13]. In adolescents, lower concentrations of adiponectin were observed in the presence of MetS [11, 14], as well as an inverse association with most of MetS components [15, 16]. However, the majority of the studies that investigated the association between adiponectin concentrations and MetS were performed in small and homogeneous samples (i.e. overweight/obese adolescents) [17, 18]. Therefore, studies addressing the relationship between adiponectin and MetS in a representative population of adolescents are scarce, especially those aiming to investigate if that association is independent of weight status [19].

Thus, in order to better understand this interaction, we developed a study based on data from the Study of Cardiovascular Risk in Adolescents (“ERICA”) to evaluate the association between adiponectin concentrations and MetS risk in a large sample of Brazilian adolescents and also to investigate if this association is independent of weight status.

## Methods

### Design and sample

ERICA is a national, school-based, cross-sectional, multicenter study conducted from 2013 to 2014, which evaluated the prevalence of cardiovascular risk factors in Brazilian adolescents (12 to 17 years old) who lived in cities with more than 100,000 inhabitants.

Sample size calculation and the sampling process have been fully described previously [20]. Briefly, the sample was divided into 32 strata, comprised of 27 capitals of Brazilian states and five more strata composed by other cities with more than 100,000 inhabitants from each of the five geographic macroregions of Brazil. Stratification was done according to three categories: schools (public or private), grade (seventh, eighth and ninth grade of Elementary and first, second and third grade of High School) and shift (morning or afternoon) of the classrooms. All adolescents from sampled classrooms were eligible to enter in the study. Sampling weight was calculated by the products of the inverse probabilities of inclusion in each selection stage and was calibrated by age and sex, considering the estimated number of adolescents from schools located in the geographic strata included in the study.

In this study, the sample consists of 4546 adolescents who attended school at morning classes in four Brazilian capitals: Brasília, Fortaleza, Porto Alegre and Rio de Janeiro. These cities are located in four of the five Brazilian macroregions, and the sample was representative at a municipality level. A full description of the study design, data collection and blood sampling is available elsewhere [21, 22].

ERICA’s protocol was approved by Research Ethics Committees in all 27 Federation units in Brazil. Adolescents were invited at schools to participate in the study, and all those enrolled agreed in writing before the data collection. In addition, we collected a written informed consent signed by the parent or legal guardian of the participant.

### Adiponectin measurement

Serum total adiponectin concentrations (µg/ml) were measured by enzyme-linked immunosorbent assay (ELISA) kit from Invitrogen^®^ (KHP0041) with a sensitivity of 0.001 µg/ml. Intra and inter-assay coefficients of variation were < 5%, following the manufacturer instructions. Laboratory analyses were performed by a single laboratory using frozen serum (− 80 °C) and following a standardized protocol [21]. Thereafter, for data analyses, adiponectin concentrations were categorized into sex and age-specific quartiles (Additional file 1: Table S1).

**Table 1:** Characteristics of study participants, ERICA 2013–2014 (n= 4546)

Characteristics	Weighted mean or frequency (95% CI)
Study centers, %	
Fortaleza	24.4 (23.1, 25.6)
Rio de Janeiro	32.8 (31.4, 34.1)
Porto Alegre	18.1 (31.4, 34.1)
Brasília	24.7 (23.5, 26.0)
Female sex, %	61.2 (59.8, 62.6)
Age, years	14.9 (14.8, 14.9)
Skin color, %	
White	39.9 (36.5, 43.4)
Black	9.2 (7.5, 11.3)
Others (mixed, native or yellow)	50.9 (48.2, 53.5)
Public School, %	66.5 (55.9, 75.6)
Socioeconomic status (tertile), %	
First (poorest)	36.9 (33.1, 40.9)
Second	27.6 (25.3, 30.1)
Third	35.5 (30.6, 40.7)
Skip breakfast*, %*	51.0 (48.2, 53.9)
Physical inactivity, %	47.5 (44.8, 50.3)
Body mass index, kg/m^2^	21.7 (21.4, 21.9)
Waist circumference, cm	72.8 (72.1, 73.6)
Systolic BP, mmHg	110.8 (110.1, 111.5)
Diastolic BP, mmHg	65.8 (65.3, 66.3)
Mean BP, mmHg	80.8 (80.2, 81.3)
HDL-c, mg/dl	47.7 (47.2, 48.3)
Triglycerides (log)^a^, mg/dl	70.3 (69.0, 71.5)
Fasting plasma glucose, mg/dl	86.9 (86.5, 87.4)
Metabolic syndrome, z-score	− 0.05 (− 0.22, 0.13)
Adiponectin^b^, µg/ml	13.4 (12.8, 14.00)

BP, blood pressure; HDL-c, high density lipoprotein cholesterol

^a^Triglycerides were log transformed and reported as geometric mean and 95% CI

^b^Adiponectin was reported as median and 95% CI

### MetS components

Waist circumference was measured using an inelastic measuring tape. The measurement was done horizontally, at half the distance between the iliac crest and the lower costal margin. Systolic and diastolic blood pressure were measured using an automatic oscillometric device (Omron^®^ 705-IT), previously validated for use in youth [20]. Three consecutive measures were taken in the student’s right arm after 5 min sitting in a quiet position, and with an interval of at least 3 min between each measure. The second and third blood pressure readings were averaged and used in the analyses.

All participants were asked to refrain from food for 10–12 h before blood sampling. Compliance with the overnight fasting was determined by questionnaire before venipuncture. Fasting blood samples were collected for measurements of glucose, high-density lipoprotein cholesterol (HDL-c) and triglycerides. All blood samples were analyzed by a single laboratory following a standardized protocol [21].

### MetS risk z-score

Before data analyses, all MetS components were standardized to the mean (z-score) by gender and age. Broadly based on the definition proposed by the International Diabetes Federation (IDF) [23], we constructed a continuously distributed MetS risk z-score (MetS-z), approach which was widely reported in the literature [24–26]. This variable was derived by standardizing and then summing the following continuously distributed MetS components: waist circumference, high blood pressure (average of systolic blood pressure and diastolic blood pressure), hyperglycemia (fasting plasma glucose) and dyslipidemias (inverted fasting HDL-c, and log-transformed triglycerides). Higher values on the MetS-z were indicative of a poorer metabolic profile and higher risk for development of MetS [24, 26].

### Covariates

The following variables were examined as covariates: study centers (Rio de Janeiro, Porto Alegre, Fortaleza and Brasília), sex, age groups (12–13, 14–15 and 16–17 years), skin color (white, black and mixed/yellow/native), and type of school (public or private). An economic index, similar to which was used in Brazilian demographic census, was used to assess economic status [27]; it considered possession of certain goods and the presence of a housekeeper at home. Thereafter, the index was categorized in tertiles.

Skipping breakfast (always/very often) was self-reported and considered an indicator of unhealthy eating habits. Time spent in moderate-to-vigorous physical activity was assessed using an adapted version of the Self-Administered Physical Activity Checklist Questionnaire [28], cross-culturally adapted and validated for Brazilian adolescents [29]. To determine the weekly amount of time spent in physical activity, we multiplied self-reported duration and frequency for each activity listed and then dichotomized it in < 300 or ≥ 300 min/week.

Trained researchers measured the clinical variables. Height was measured twice using a portable stadiometer with a 0.1 variation, and the mean of the two values obtained was considered in the analyses. Weight was measured using a digital scale in light clothing. Body mass index (BMI) was calculated using the standardized formula [BMI = weight (kg)/height^2^(m^2^)]. The World Health Organization reference curves were used to classify adolescents with normal weight (BMI z-score ≤ 1), overweight (BMI z-score > 1 and ≤ 2) and obesity (BMI z-score > 2) [30]. Overweight and obesity were then combined into one category.

### Statistical analysis

Fasting triglycerides were logarithmically transformed owing to their skewed distributions (geometric mean and 95% confidence intervals (95% CI) are presented in the results). Adiponectin concentrations were described by median values and 95% CI. Thereafter, this variable was categorized according to sex and age-specific quartiles (Additional file 1: Table S1).

Initially, we assessed the distribution of investigated cardio-metabolic risk factors according to the quartiles of adiponectin, and the linear trends were evaluated by Wald’s test. Associations between quartiles of adiponectin and MetS-z and MetS components were investigated by multiple linear regressions. Our first model was adjusted for sex, age, skin color and socioeconomic status. These variables were kept in the second model in addition to physical activity and skipping breakfast. In the final adjusted model, BMI was included as a potential confounder (adiposity-adjustment).

The global adjustment of the models was evaluated. We did not find evidence of multicollinearity into the models, even when BMI was included. The adiponectin and sex interaction for the association with MetS-z and MetS components was tested using a multiplicative approach. In further analyses, we investigated the association between quartiles of adiponectin and MetS-z after stratifying the sample for weight status, considering the possibility that this association could be modified in the presence of overweight/obesity. We also calculated a MetS-z score without the adiposity component (i.e., waist circumference) to examine whether the association between the main exposure and MetS-z is mediated by adiposity.

To obtain population-representative findings, analyses were conducted using sample weights for ERICA, which accounted for the complex survey design [20]. All tests were two-tailed. The analyses were performed using Stata version 14 (StataCorp, College Station, TX, USA).

## Results

The analysed sample was composed of 4546 adolescents. Table 1 shows the main characteristics of the sample. Most adolescents were female and students from public schools. The mean age was 14.9 (SD = 1.5) years old. Overall, 19.1% and 9.1% of the adolescents were categorized as overweight and obese, respectively. According to the IDF criteria for youth, the prevalence of metabolic syndrome in this sample was 2.0% (95% CI 1.6%, 2.5%), similar in boys and girls.

The median of adiponectin was lower in those with overweight/obesity (11.6 µg/ml; 95% CI 10.6, 12.6) compared to those with normal weight (14.3 µg/ml, 95% CI 13.6, 15.0). The means of MetS-z were − 1.1 (95% CI − 1.2, − 0.9) and 2.3 (95% CI 2.0, 2.6) in adolescents with normal weight and overweight/obesity, respectively.

We did not find any indicative of interaction between sex and adiponectin concentrations for the association with MetS-z (*p* value for interaction > 0.8) or MetS components (p-value for interaction > 0.5), thus all analyses were performed for the overall sample. The values of sex and age-specific quartiles of adiponectin are presented in the Additional file 1: Table S1. The cut-off points for those in the reference group (1st quartile) were lower among boys compared with girls and decreased with age in both sexes.

Table 2 shows the means of the MetS-z and MetS components according to the quartiles of adiponectin. Lower values of waist circumference, triglycerides and MetS-z, as well as increased HDL-c concentrations were observed in the upper quartiles of adiponectin. However, no variations in blood pressure or fasting plasma glucose values were observed across quartiles of adiponectin.

**Table 2:** Mean and 95% CI of metabolic syndrome risk score and its individual components according to quartiles of adiponectin, ERICA 2013–2014

	Quartiles of adiponectin				p for trends
	First (n = 1092)	Second (n = 1101)	Third (n = 1112)	Fourth^b^ (n = 1241)	
Waist circumference, cm	74.7 (73.8, 75.6)	73.5 (73.4, 75.6)	72.2 (71.2, 73.3)	70.9 (70.1, 71.7)	< 0.001
Systolic BP, mmHg	111.3 (110.2, 112.4)	110.8 (109.6, 112.1)	111.0 (109.8, 112.2)	110.1 (109.1, 111.1)	0.157
Diastolic BP, mmHg	65.8 (65.0, 66.6)	65.9 (65.1, 66.7)	65.9 (65.1, 66.7)	65.5 (64.9, 66.2)	0.622
Mean BP, mmHg	81.0 (80.1, 81.8)	80.9 (80.0, 81.8)	80.9 (80.0, 81.8)	80.4 (79.6, 81.1)	0.334
Triglycerides^a^ (log), mg/dl	71.9 (69.5, 74.3)	72.1 (69.4, 74.9)	70.1 (67.7, 72.6)	67.1 (65.1, 69.1)	0.004
HDL-c, mg/dl	46.6 (45.9, 47.3)	46.3 (45.5, 47.1)	48.1 (47.2, 49.1)	50.0 (48.9, 50.9)	< 0.001
Fasting plasma glucose, mg/dl	87.2 (86.4, 88.0)	87.1 (86.4, 87.8)	86.9 (86.2, 87.6)	86.5 (85.9, 87.2)	0.222
MetS-z, z-score	0.35 (0.14, 0.57)	0.24 (− 0.04, 0.53)	− 0.12 (− 0.41, 0.18)	− 0.66 (− 0.89, − 0.43)	< 0.001
MetS-z without waist circumference, z-score	0.15 (− 0.03, 0.34)	0.18 (− 0.06, 0.42)	− 0.06 (− 0.28, 0.16)	− 0.46 (0.64, − 0.28)	< 0.001

BP, blood pressure; HDL-c, high density lipoprotein cholesterol; MetS-z, metabolic syndrome risk z-score

^a^Triglycerides was log transformed and reported as geometric mean and 95% CI

^b^Highest values

Table 3 shows the associations between adiponectin levels, MetS-z and MetS components. In the first (sociodemographic-adjusted) and second (behavior-adjusted) models, adiponectin concentrations were inversely associated with waist circumference, triglycerides and MetS-z. In addition, HDL-c concentrations increased through the quartiles of adiponectin. After including BMI in the model (adiposity-adjusted) and despite a reduction in the strength of the associations, the coefficients remained significant for HDL-c, waist circumference and MetS-z for those adolescents in the top quartile of adiponectin. In sensitivity analysis, waist circumference was removed from the outcome to investigate the possibility that obesity may drive the observed association. The associations were somewhat attenuated, but results were materially unchanged.

**Table 3:** Association between quartiles of adiponectin with metabolic syndrome risk score and its individual components, ERICA 2013–2014

Clinical outcomes	Quartiles of adiponectin							p for trends
	First (n = 1092)	Second (n = 1101)		Third (n = 1112)		Fourth (n = 1241)^b^		
	*β* (95% CI)							
Model 1: adjusted for sex, age, skin color and socioeconomic status								
Waist circumference, cm	Ref	− 1.65	(− 2.98, − 0.31)	− 3.04	(− 4.51, − 1.56)	− 3.83	(− 5.20, − 2.46)	< 0.001
Systolic BP, mmHg	Ref	− 1.04	(− 2.29, 0.21)	− 0.75	(− 2.05, 0.55)	− 1.16	(− 2.70, 0.37)	0.220
Diastolic BP, mmHg	Ref	− 0.07	(− 0.97, 0.84)	− 0.10	(− 1.08, 0.88)	− 0.31	(− 1.33, 0.71)	0.586
Mean BP, mmHg	Ref	− 0.39	(− 1.33, 0.55)	− 0.32	(− 1.35, 0.71)	− 0.60	(− 1.72, 0.53)	0.381
Triglycerides (log)^a^, mg/dl	Ref	0.001	(− 0.05, 0.05)	− 0.03	(− 0.07, 0.02)	− 0.07	(− 0.12, − 0.02)	0.002
HDL-c, mg/dl	Ref	− 0.43	(− 1.57, 0.71)	1.34	(0.15, 2.53)	3.05	(1.83, 4.26)	< 0.001
Fasting plasma glucose, mg/dl	Ref	0.16	(− 0.65, 0.97)	− 0.10	(− 0.84, 0.64)	− 0.43	(− 1.46, 0.59)	0.308
MetS-z, z-score	Ref	− 0.16	(− 0.45, 0.14)	− 0.55	(− 0.86, − 0.24)	− 0.98	(− 1.27, − 0.69)	< 0.001
MetS-z without waist circumference, z-score	Ref	0.22	(− 0.25, 0.29)	− 0.22	(− 0.49, 0.05)	− 0.57	(− 0.82, − 0.32)	< 0.001
Model 2: adjusted for variables in model 1 plus physical activity and skipping breakfast								
Waist circumference, cm	Ref	− 1.94	(− 3.36, − 0.53)	− 3.28	(− 4.84, − 1.73)	− 4.07	(− 5.56, − 2.58)	< 0.001
Systolic BP, mmHg	Ref	− 1.20	(− 2.47, 0.07)	− 0.86	(− 2.22, 0.51)	− 1.39	(− 3.02, 0.24)	0.165
Diastolic BP, mmHg	Ref	− 0.17	(− 1.13, 0.79)	− 0.09	(− 1.09, 0.91)	− 0.48	(− 1.56, 0.60)	0.472
Mean BP, mmHg	Ref	− 0.51	(− 1.49, 0.47)	− 0.35	(− 1.41, 0.72)	− 0.78	(− 1.98, 0.41)	0.293
Triglycerides (log)^a^, mg/dl	Ref	− 0.01	(− 0.06, 0.04)	− 0.04	(− 0.09, 0.01)	− 0.08	(− 0.12, − 0.0− 3)	0.001
HDL-c, mg/dl	Ref	− 0.23	(− 1.40, 0.95)	1.87	(0.59, 3.15)	3.02	(1.77, 4.27)	< 0.001
Fasting plasma glucose, mg/dl	Ref	− 0.05	(− 0.92, 0.82)	− 0.44	(− 1.28, 0.39)	− 0.62	(− 1.65, 0.41)	0.143
MetS-z, z-score	Ref	− 0.29	(− 0.60, 0.02)	− 0.71	(− 1.06, − 0.37)	− 1.07	(− 1.38, − 0.75)	< 0.001
MetS-z without waist circumference, z-score	Ref	− 0.08	(− 0.36, 0.19)	− 0.36	(− 0.65, − 0.07)	− 0.63	(− 0.88, − 0.38)	< 0.001
Model 3: adjusted for variables in models 2 plus body mass index								
Waist circumference, cm	Ref	− 0.42	(− 0.97, 0.14)	− 0.27	(− 0.86, 0.31)	− 0.81	(− 1.52, − 0.11)	0.030
Systolic BP, mmHg	Ref	− 0.28	(− 0.58, 1.01)	0.96	(− 0.28, 2.20)	0.59	(− 0.78, 1.95)	0.218
Diastolic BP, mmHg	Ref	0.23	(− 0.72, 1.17)	0.68	(− 0.21, 1.57)	0.37	(− 0.62, 1.37)	0.373
Mean BP, mmHg	Ref	0.05	(− 0.92, 1.02)	0.78	(− 0.15, 1.71)	0.44	(− 0.61, 1.50)	0.275
Triglycerides (log)^a^, mg/dl	Ref	0.001	(− 0.50, 0.05)	− 0.01	(− 0.06, 0.04)	− 0.04	(− 0.09, 0.001)	0.049
HDL-c, mg/dl	Ref	− 0.60	(− 1.79, 0.60)	1.11	(− 0.24, 2.46)	2.22	(0.98, 3.47)	< 0.001
Fasting plasma glucose, mg/dl	Ref	0.03	(− 0.85, 0.91)	− 0.28	(− 1.13, 0.58)	− 0.43	(− 1.49, 0.63)	0.318
MetS-z, z-score^c^	Ref	0.02	(− 0.25, 0.30)	− 0.10	(− 0.38, 0.18)	− 0.40	(− 0.66, − 0.14)	0.005
MetS-z without waist circumference, z-score^c^	Ref	0.10	(− 0.20, 0.39)	− 0.04	(− 0.33, 0.25)	− 0.28	(− 0.53, − 0.03)	0.031

BP, blood pressure; HDL-c, high density lipoprotein cholesterol; MetS-z, metabolic syndrome risk z-score

^a^Triglycerides was log transformed

^b^Highest values

^c^Mean of MetS-z: − 0.05 (SE 0.09); mean of MetS-z without waist circumference: − 0.04 (SE 0.06), p for heterogeneity < 0.001

**Fig. 1:**
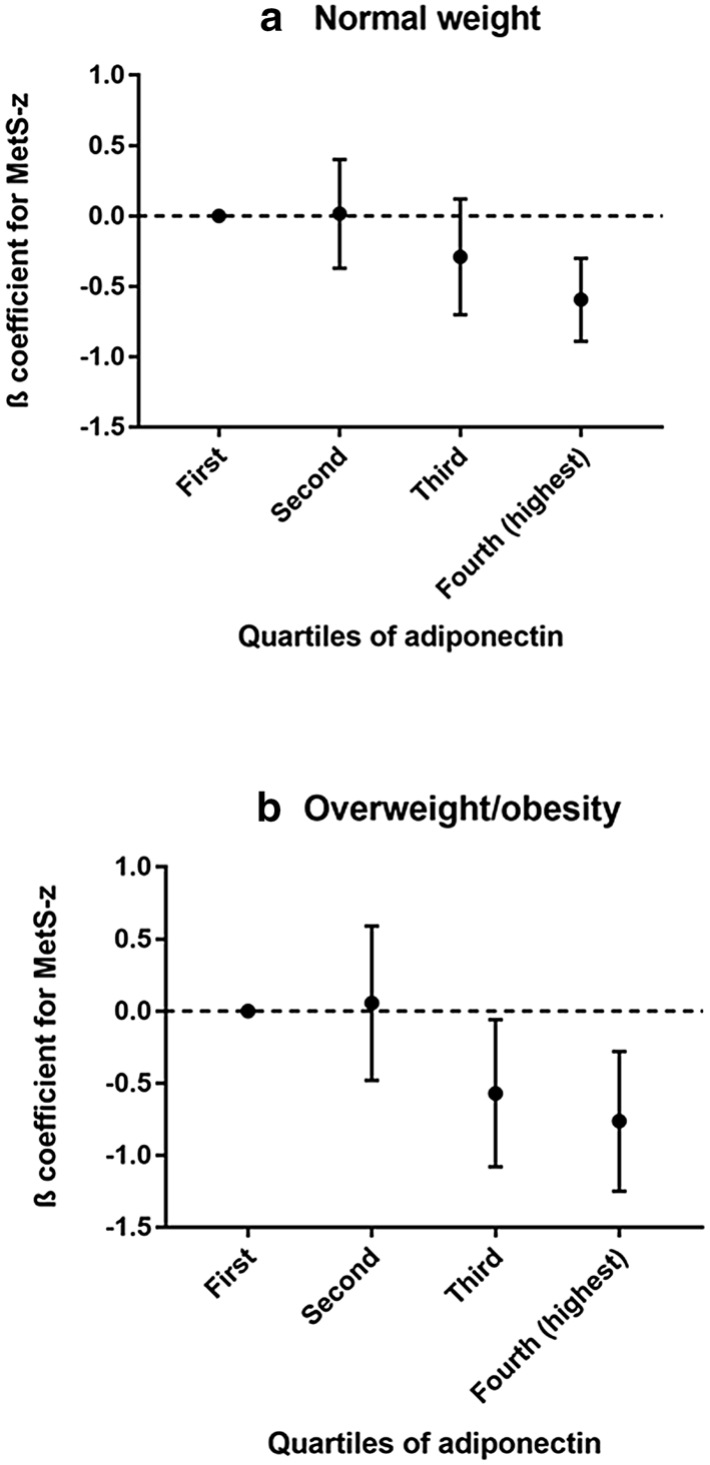
Associations between adiponectin concentration and metabolic syndrome z-scored in normal weight (**a**) and overweight/obesity (**b**) adolescents. ERICA 2013–2014

The associations between quartiles of adiponectin and the MetS-z according to weight status are presented in Fig. 1. The MetS-z was reduced in higher quartiles of adiponectin, in both normal weight and overweight/obese adolescents. However, the association observed seems to be slightly more pronounced among adolescents with overweight/obesity (Panel b) when compared with normal weight youth (Panel a).

## Discussion

In this study, we aimed to evaluate the association between adiponectin concentrations and MetS-z. Our findings showed a significant and inverse association between higher levels of adiponectin and MetS-z independently of potential confounders, including BMI, in Brazilian adolescents. Additionally, after splitting the sample by weight status, the association between adiponectin and MetS-z remained unchanged in both normal weight and overweight/obese adolescents.

The role of adiponectin in the pathogenesis of MetS remains controversial. Other authors have hypothesized that adiponectin concentrations are influenced by several traditional risk factors for Mets (i.e. sex, age, diet, physical activity, etc.), and that those risk factors may induce adiponectin resistance. Abnormal concentrations of adiponectin are related to low grade inflammation, which represents a key factor in the development of MetS, and it is directly associated with insulin resistance and regulation of adipose tissue distribution [7].

Regarding each individual component of MetS, in our models adjusted for sociodemographic and behavioral characteristics, adiponectin concentrations were positively associated with HDL-c and inversely associated with waist circumference and triglycerides. After adjusting for BMI, only the association with triglycerides overlapped the reference value. In accordance to the literature, adiponectin seems to act on reduction of triglycerides concentrations, increasing glucose uptake by the skeletal muscle and thus increasing HDL-c through hepatic lipase activity [31]. Previous study with adolescents also showed correlations between adiponectin and HDL-c, triglycerides and blood pressure [14, 32, 33].

In this study the concentrations of adiponectin were inversely associated with MetS-z even after adjustment for BMI, although not without attenuation in strength of the association. To use of MetS-z instead of dichotomous criteria can be powerful, however it is difficult to interpret whether changes in β coefficients are clinically meaningful. However, if we consider that all cardio-metabolic risk factors involved in MetS-z are continuously associated with cardiovascular risk and that a good health is expected in this age group, it is possible to consider that any significant change in β coefficients are relevant in terms of public health.

The association between adiponectin concentrations and MetS has been described before, especially in overweight populations (24–26), but only a few studies have investigated whether this association is independent or not of adiposity [32, 34]. In a Mediterranean pediatric cohort (n = 1138), adiponectin was no longer significantly associated with higher number of MetS components after adjustment for BMI, although the prevalence of MetS in this study was very low (0.7%) and was only observed in youth with obesity [32]. On the *Identification and prevention of Dietary and lifestyle induced health Effects In Children and infants study* (IDEFICS), after inclusion of BMI in the model, the association between adiponectin and MetS was no longer observed, suggesting that this relation may be mediated by adiposity [11]. Finally, other authors observed that low concentrations of adiponectin were prospectively associated with a poor cardiometabolic profile, but only among overweight youth [35].

On the other hand, adiponectin was associated with MetS in Chinese adolescents [15] and with several MetS components in a sample of Puerto Rican youth [36], and, in both studies, these results were independent of adiposity. These data are in line with our results, suggesting that adiponectin may play a role in the prevention of MetS. Our study extends these previous observations while including a large and representative sample of a multiethnic population of adolescents. In sensitivity analysis, waist circumference was removed from the outcome to investigate the possibility that obesity may drive the observed association; however, it was only partially confirmed because the association was attenuated but did not disappear. The ERICA sample size also allowed us to stratify the sample by weight status, and after that, results regarding the association between adiponectin and MetS-z were materially unchanged.

Our work has some particular observations. We calculated the quartiles of adiponectin adjusted by age and sex, in part because there is not a well-established cutoff point for unhealthy concentrations of adiponectin. To our knowledge, only two previous studies suggested an optimal cutoff point of adiponectin to detect MetS in adolescents, however both samples were composed of only obese adolescents [16, 37]. We also calculated sex and age-specific continuous MetS z-scored like others [25, 26, 38], because this increases statistical power. It is a concern in studies with apparently healthy adolescents since the prevalence of MetS is very low, considering dichotomized definitions, and MetS z-scored uses full information from the cardio-metabolic risk factors evaluated [39].

Potential limitations of a cross-sectional study include temporal bias. Some of the covariates investigated were self-reported, which can introduce information bias. Furthermore, our analyses were based on a single evaluation of serum adiponectin concentrations, which may increase random measurement error and, consequently, underestimate the observed association. Also, high-molecular weight adiponectin has been suggested to be a better predictor of cardio-metabolic parameters than total adiponectin [40, 41], which could have contributed for more conservative results in this study. Finally, adiponectin is one of many biochemical variables related with pro- and anti-inflammatory processes that are produced by adipocytes, and it is not produced equally in all adipose tissues. Furthermore, fat distribution can also interfere in the association of adiponectin with cardio-metabolic outcomes [42, 43].

However, although having some limitations, we believe that our study has several strengths. Most notably, it includes a large, representative and multiethnic sample of adolescents from a developing country. We corrected all analyses for the complex sampling design and adjusted it for a number of potentially important confounding factors, including BMI. In addition, the sample size allowed us to stratify the analyses by weight status. Finally, all biochemical variables, including serum adiponectin concentrations and MetS components, were analyzed using standardized procedures in one central laboratory.

In conclusion, the present results suggests that increased adiposity mediates the association between adiponectin and MetS during adolescence, and this association is attenuated after adjustments for BMI. However, we extended this knowledge using data from a well-developed and designed study, which shows that higher adiponectin concentrations are independently associated with MetS risk in Brazilian adolescents, even after adjusting for BMI. These results were similar in normal weight and overweight/obese adolescents, suggesting that adiponectin may play a role in early development of MetS independently of adiposity.

## Additional file

**Additional file 1: Table S1.** Values of sex and age-specific quartiles of adiponectin. ERICA 2013–2014 (n = 4546).

## Data Availability

The datasets generated and/or analyzed during the current study are not publicly available due to issues in making them accessible online, such as storage difficulties. However, the datasets are available on reasonable request to the corresponding author.

## Abbreviations

MetS: metabolic syndrome
ERICA: Study of Cardiovascular Risk in Adolescents
ELISA: enzyme-linked immunosorbent assay
HDL-c: high-density lipoprotein cholesterol
BMI: body mass index
MetS-z: metabolic syndrome risk z-score
