# Supplementary material for: Higher adiponectin concentrations are associated with reduced metabolic syndrome risk independently of weight status in Brazilian adolescents
Source: Diabetol Metab Syndr. 2019 May 24;11:40. doi: 10.1186/s13098-019-0435-9 (PMC6534928; doi:10.1186/s13098-019-0435-9)
Supplement: Supplementary file 1 — Additional file 1: Table S1. Values of sex and age-specific quartiles of adiponectin. ERICA 2013–2014 (n = 4546). [file 13098_2019_435_MOESM1_ESM.docx]

| **Additional Table S1.** Values of sex and age-specific quartiles of adiponectin. ERICA 2013-2014 (n=4,546). | | | | | |
| --- | --- | --- | --- | --- | --- |
| **Sex** | **Age (years)** | **Adiponectin (µg/ml) - quartiles** | | | |
|  |  | **< 25^th^** | **≥ 25^th^ and < 50^th^** | **≥ 50^th^ and < 75^th^** | **≥ 75^th^** |
| Female | 12-13 | 9.20 | 9.20 - 14.42 | 14.42 - 20.16 | 20.16 |
|  | 14-15 | 8.29 | 8.29 - 13.74 | 13.74 - 20.31 | 20.31 |
|  | 16-17 | 8.20 | 8.20 - 14.03 | 14.03 - 20.92 | 20.92 |
| Male | 12-13 | 7.27 | 7.27 - 12.85 | 12.85 - 19.49 | 19.49 |
|  | 14-15 | 7.00 | 7.00 - 12.50 | 12.50 - 18.78 | 18.78 |
|  | 16-17 | 6.88 | 6.88 - 12.82 | 12.82 - 18.89 | 18.89 |
